# Fish intake reflects on DHA level in breast milk among lactating women in Latvia

**DOI:** 10.1186/s13006-018-0175-8

**Published:** 2018-07-20

**Authors:** Līva Aumeistere, Inga Ciproviča, Dace Zavadska, Viktors Volkovs

**Affiliations:** 1 Faculty of Food Technology, Latvia University of Life Sciences and Technologies, Jelgava, Latvia; 2 Institute of Food Safety, Animal Health and Environment BIOR, Riga, Latvia; 3 Department of Paediatrics, Riga Stradiņš University, Riga, Latvia

**Keywords:** Breast milk, Docosahexaenoic acid, Lactation, Breastfeeding, Dietary habits, Maternal nutrition, Marine food

## Abstract

**Background:**

Docosahexaenoic acid (DHA) is an essential fatty acid required for proper growth and development. DHA levels in breast milk vary worldwide. Higher levels are observed among coastal populations and are associated with marine food consumption. Latvia is located in Northern Europe, on the eastern shore of the Baltic Sea. Nevertheless, fish consumption among women of reproductive age is low. The aim of this study was to determine DHA levels in breast milk among lactating women in Latvia.

**Methods:**

Invitation to participate in the study was posted on a social media member group for breastfeeding mothers. In total, 71 women were enrolled from November 2016 until December 2017. DHA levels (% of total fatty acids) in breast milk were measured by gas chromatography. Information about food consumed during the three consecutive days prior to the milk sampling was obtained and a food frequency questionnaire (FFQ) was completed. Information about maternal and infant characteristics, current breastfeeding pattern and milk expression method was also collected.

**Results:**

The mean age of participants was 31 ± 4 years and the mean BMI was 22.1 ± 3.2. 27 participants were primiparas. The average age and birth weight for infants (34 males, 33 females) was 6 ± 4 months and 3.46 ± 0.55 kg, respectively. The median DHA level in breast milk (*n* = 60) was 0.30 ± 0.18% of total fatty acids and it was not influenced by any of the maternal or infant’s characteristics nor current breastfeeding pattern or milk expression manner (*p* > 0.05). Fish intake was a positive predictor for DHA levels in breast milk (*r* = 0.318, *p* = 0.013). Average maternal DHA intake was 136 ± 26, 137 ± 33 and 178 ± 49 (SEM – standard error of the mean) mg, for the third, second and last day prior to sampling day, respectively.

**Conclusions:**

DHA levels in breast milk among lactating women in Latvia correspond to the suggested target DHA value in breast milk (0.30%). Fish consumption is a significant positive predictor for DHA levels in breast milk, however, daily DHA intake among the participants was lower than recommended (200 mg).

## Background

Breast milk gives infants most of the nutritional needs required for proper growth and development. Mothers are encouraged to exclusively breastfeed for the first six months and thereafter infants should receive complementary foods with continued breastfeeding for up to two years of age and beyond [1]. Docosahexaenoic acid (DHA) (C22:6 n - 3) is a long chain polyunsaturated fatty acid that is an important component of retinal photoreceptors, as well as brain cell membranes. Therefore, DHA is essential for visual and cognitive development [2–8] and DHA status in infancy may fundamentally impact long-term health [6]. The European Food Safety Authority (EFSA) recommends 100 mg per day as the adequate intake of DHA for infants and young children (024 months old) [9].

As the synthesis of DHA is limited in human cells, breast milk serves as a DHA source for infants. Breast milk DHA may derive either from mother’s diet, body stores or be endogenously synthesized from the precursor α-linolenic acid (18:3 n - 3) (ALA) in maternal tissues [2]. However, it seems that the ability to convert ALA to DHA is low [10, 11] and correspondingly, maternal dietary ALA intake does not significantly affect DHA levels in breast milk [2, 12].

Fortunately, dietary DHA is well absorbed and transferred to breast milk [2, 13]. Fish and other marine foods, as well as microalgae oil contain higher amounts of DHA than other animal origin products (eggs and meat) [2, 6, 7].

Maternal intake of DHA during lactation should be at least 200 mg per day [3, 4]. Women can meet the recommended intake by consuming one-to-two portions of fatty fish per week (equivalent to 150–300 g). EFSA states that breastfeeding women can select fish from a wide range of species but advises to avoid large predatory fish such as swordfish and tuna, as these fish are likely to contain higher levels of methylmercury [14]. Dietary supplements containing DHA is an option for women who do not eat enough DHA-rich foods [6, 9, 15].

Breast milk composition is constantly changing and dietary fatty acids are rapidly transported from chylomicrons into breast milk with a peak between six and twelve hours after dietary DHA intake [15, 16]. Furthermore, it should be noted that DHA levels in breast milk can stay significantly elevated for two days after the dietary intake [15, 16].

Although maternal diet seems to be the most important variable affecting DHA levels in breast milk, other characteristics like parity can also be influencing factors [17, 18].

Breast milk fatty acid composition has been studied throughout the world and the mean DHA level in mature breast milk, summarizing the data from different countries, is 0.32 ± 0.22% of total fatty acids [18–25]. Higher DHA levels in breast milk (exceeding 1% of total fatty acids) are seen among coastal populations and are associated with marine food consumption [19, 26]. However, there are currently no studies about fatty acid composition among lactating women in Latvia. Latvia is located in Northern Europe, on the eastern shores of the Baltic Sea, nevertheless, fish consumption is a nutritional problem for the population of Latvia. According to health behaviour among the Latvian adult population survey data of 2016, fish consumption frequency is low for women of reproductive age (15 to 44 years) [27]. The survey data indicated that approximately half of the women (49%) were non-fish eaters and only 42% consumed fish one-to-two times a week [27].

The aim of this study was to determine DHA levels in breast milk among lactating women in Latvia.

## Methods

### Participants

Invitation to take part in the study was posted on a social media member group for breastfeeding mothers. In total, 71 women were enrolled in this cross-sectional study from November 2016 until December 2017. The inclusion criteria for participants were: (1) currently living in Latvia; (2) without metabolic disorders; (3) singleton pregnancy; (4) infant at least one month old; (5) currently exclusively breastfeeding or partially breastfeeding (breast milk and infant formula and/or complementary food).

### Data collection

Participants were asked to write each foodstuff and dietary supplement they had consumed in the three consecutive days prior to the sampling day. Participants were allowed to consume self-chosen diets. No dietary recommendations were given before the study. Size measures (spoons and measuring cups) and a photographic atlas of food portions [28] were used to help complete a food diary.

In addition, habitual intake of DHA was assessed using a food frequency questionnaire (FFQ) – a transformed blank taken from guidelines developed by the World Health Organization [29]. The FFQ contained information about DHA sources like fish, crustaceans, eggs and meat (beef, pork, poultry and turkey) and their consumption frequency. Also, information about the consumption frequency of other fat sources – milk and dairy products, margarine, fat spreads, vegetable oils – as well as other plant based products (avocado, nuts and seeds) in the last month prior to study was obtained. The response options were arranged in six categories from “never”, “less than once a week”, “once a week”, “twice a week”, “more than twice a week but not every day” to “every day”.

Information about maternal sociodemographic variables (maternal age, body mass index (BMI), parity, sex and age of infant) was collected. Maternal BMI was calculated based on self-reported information about weight and height. Anthropometric measurements were not made during this study. Participants were also asked to specify their current breastfeeding manner (exclusive or partial) and the milk expression method used during the study (by hand, breast pump or a combination of both).

Four of the 71 participants were excluded from the study at this stage because they did not complete the food diary, FFQ and/or information about the above mentioned sociodemographic variables.

### Sample collection and analysis

Participants were instructed to obtain pooled milk samples (~ 10 ml) within a 24-h period. A few millilitres of milk were to be expressed after the end of nursing from the feeding breast, thus providing hindmilk for the study. Sampling frequency was not specified, however, taking into account possible diurnal variations, participants were asked to include milk from morning, mid-day and evening feedings. Mothers were allowed to use the most convenient milk expression method during the study. Samples were collected into pre-labelled polypropylene containers provided to each participant. Mothers were instructed to store milk in the refrigerator (4 ± 2 °C) during the collection process, then once completed place the container in the freezer (-15…-18 °C). Samples were transported to the laboratory in a cooler with ice packs and kept frozen at -18 ± 3 °C until analysis.

Fatty acids were analysed by gas chromatography with flame ionization detection after solvent extraction and transesterification of fats with methanol. Samples (5.00 g) were weighed in 50 mL polypropylene test tubes with screw caps and extracted with 20 mL of acetone/hexane solution (1:1). The mixture was centrifuged (3500 rpm, 10 min). The organic layer was transferred into a 15 mL polypropylene screw cap test tube and evaporated with nitrogen (55 °C). 100 μL of concentrated residue was transferred into a 22 mL glass tube and dissolved in 2 mL of isooctane, then 200 μL of sodium methoxide was added and the solution was mixed twice using a vortex mixer. After, the solution was extracted with 2 mL of sodium chloride (40%). 10 μL of the organic layer was transferred into 250 μL autosampler vials and diluted with 190 μL of cyclohexane. Fatty acid methyl esters were analysed using a GC-FID instrument (Agilent; 6890 N) equipped with an autosampler (Agilent; 7683 Series). Aliquots of the fatty acid methyl esters (2 μL) were injected at a 10:1 split ratio and at 280 °C into a BPX70 capillary column (30 m × 0.32 mm BPX70 phase, 0.25 μm film thickness). The flow rate of the helium carrier gas was 1.0 mL min^- 1^. The oven temperature programs were set as follows. The initial temperature of 80 °C was held for two minutes, then increased to 130 °C at 50 °C min^- 1^ and held for ten minutes, then increased to the final temperature of 180 °C at 2 °C min^- 1^ and held for another ten minutes. The detector temperature was 300 °C. Fatty acid methyl esters were identified, comparing their relative retention times with authentic standards (Supelco 37 component FAME mix, Supelco Inc.). The sum of all fatty acid peak areas was calculated and the relative proportion of each fatty acid was determined, expressing it as mass ratio from the sum of all peaks (%).

### Statistical methods

Total dietary DHA intake was calculated using the United States Department of Agriculture’s (USDA) Branded Food Products Database [30]. Data about DHA content of different dietary supplements was taken from the manufacturer’s website. Data statistical analysis was performed using the software - IBM SPSS Statistics, version 22.0 (Spss Inc., Chicago, IL, USA). Spearman’s correlation coefficient was used to assess how the mother’s and infant’s age, maternal BMI, parity and dietary habits affects DHA levels in breast milk. The Kruskal-Wallis test was used to evaluate if milk expression manner, infant’s sex or feeding pattern affect DHA levels in breast milk. The Kruskal-Wallis test was also used to evaluate if there is a difference in DHA levels in breast milk among non-fish eating mothers and participants eating fish once or ≥ 2 a week. Linear regression was performed to predict the DHA level in breast milk based on fish intake data from the FFQ. Statistical significance was defined as *p* <  0.05.

## Results

### Characteristics of participants

The mean age for participants was 31 ± 4 years and the mean BMI was 22.1 ± 3.2. The majority of the women were primiparous (*n* = 27). The average age and birth weight for infants was 6 ± 4 months and 3.46 ± 0.55 kg, respectively (Table 1).

**Table 1:** Descriptive characteristics of participants (*n* = 67)

Characteristics	Mean ± SD	Range
Maternal characteristics		
Age (years)	31 ± 4	23–39
Body mass index (BMI)	22.1 ± 3.2	17.6–32.2
Parity	27 primiparas, 40 multiparas	
Infants’ characteristics		
Age (months)	6 ± 4	1.5–21
Birth weight (kg)	3.46 ± 0.55	1.60–4.77
Sex	33 females, 34 males	

For milk expressions, participants most commonly chose a breast pump (*n* = 37), followed by manual expression (*n* = 21) and then a combination of both methods (*n* = 9).

### Breast milk fatty acid profile

The fatty acid profile is shown in Table 2. The relative proportion of saturated, monounsaturated and polyunsaturated fatty acids in breast milk was 46.15 ± 5.64%, 38.80 ± 3.73% and 14.10 ± 5.31%, respectively. The predominant fatty acids in breast milk were palmitic acid (24.05 ± 3.49%), oleic acid (34.50 ± 3.50%) and linoleic acid (LA) (11.50 ± 4.43%).

**Table 2:** Fatty acid profile of mature breast milk (*n* = 60)

Fatty acids	Median^a^ ± SD^b^, %	Range, %
Saturated fatty acids (SFA)		
C6:0	0.10 ± 0.05	0.06–0.20
C8:0	0.10 ± 0.05	0.10–0.27
C10:0	1.05 ± 0.25	0.50–1.60
C11:0	0.10 ± 0.05	< 0.10–0.20
C12:0	4.45 ± 1.39	2.20–8.50
C13:0	0.10 ± 0.05	< 0.1–0.20
C14:0	6.55 ± 1.65	3.40–10.50
C15:0	0.40 ± 0.18	0.10–0.90
C16:0	24.05 ± 3.49	13.20–30.60
C17:0	0.30 ± 0.11	0.10–0.50
C18:0	7.70 ± 1.56	3.30–10.50
C20:0	0.10 ± .07	0.10–0.40
C21:0	0.10 ± 0.06	0.10–0.27
C22:0	0.10 ± 0.05	< 0.10–0.10
C23:0	0.10 ± 0.13	0.10–0.79
C24:0	< 0.10 ± 0.01	< 0.10–0.10
Total SFA	46.15 ± 5.64	27.20–55.60
Monounsaturated fatty acids (MUFA)		
C14:1	0.30 ± 0.12	0.10–0.60
C15:1	0.10 ± 0.05	< 0.10–0.30
C16:1	1.90 ± 0.53	1.10–3.50
C17:1	0.14 ± 0.06	0.10–0.30
C18:1 n - 9, *cis*	34.50 ± 3.50	26.30–41.40
C18:1 n - 9, *trans*	< 0.10 ± 0.00	< 0.10
C18:1 n - 11, *trans*	1.20 ± 0.37	< 0.10–1.80
C20:1	0.40 ± 0.11	0.10–0.80
C22:1	0.10 ± 0.08	< 0.10–0.50
C24:1	0.20 ± 0.06	0.10–0.40
Total MUFA	38.80 ± 3.73	29.20–45.60
Polyunsaturated fatty acids (PUFA), n - 6		
C18:2 *cis* (LA)^b^	11.50 ± 4.43	6.30–26.50
C18:2 *trans*	0.20 ± 0.22	< 0.10–1.50
C18:3	0.10 ± 0.02	< 0.10–1.10
C20:2	0.20 ± 0.08	0.20–0.50
C20:3	0.20 ± 0.09	0.10–0.40
C20:4 (ARA)^b^	0.30 ± 0.10	0.10–0.50
C22:2	0.10 ± 0.05	< 0.10–0.30
Total PUFA, n - 6	12.50 ± 4.50	7.20–27.50
Polyunsaturated fatty acids (PUFA), n - 3		
C18:3 (ALA)^b^	1.00 ± 1.40	0.50–9.60
C20:3	0.10 ± 0.06	< 0.10–0.30
C20:5	0.10 ± 0.05	< 0.10–0.10
C22:6 n (DHA)^b^	0.30 ± 0.18	0.10–0.90
Total PUFA, n - 3	1.50 ± 1.44	0.90–10.30
Total PUFA	14.10 ± 5.31	8.20–31.70
Ratio		
n - 6:n - 3	7.32 ± 4.07	2.08–22.80
LA:ALA	9.92 ± 6.44	2.14–42.60
DHA:LA	0.03 ± 0.02	0.01–0.09
ARA:DHA	1.00 ± 0 .64	0.29–4.00

^a^data are expressed as relative proportion of each fatty acid (% of total fatty acids)

^b^*SD* standard deviation, *LA* linoleic acid, *ARA* arachidonic acid, *ALA* α-linolenic acid, *DHA* docosahexaenoic acid

Seven of the 67 breast milk sample DHA determinations were considered extreme outliers – exceeding more than three times the interquartile range above the third quartile (75^th^ percentile) – and were removed from further data processing. After the removal of extreme outliers, DHA levels in breast milk varied from 0.10 to 0.90% with a median level 0.30 ± 0.18% of total fatty acids.

Ratio for n - 6 versus n - 3 fatty acids was ~ 7:1. The ratio for LA and ALA was ~ 10:1. The ratio for DHA and LA was 0.03:1 but the ratio for arachidonic acid (ARA) and DHA was 1:1.

No significant correlation was found between DHA levels and dominating fatty acids (palmitic acid: *r* = -0.088, *p* = 0.504; oleic acid: *r* = -0.026, *p* = 0.846; LA: *r* = -0.104, *p* = 0.429) in breast milk. No significant correlation was also found regarding DHA and ALA (*r* = 0.047, *p* = 0.722) or ARA (*r* = 0.039, *p* = 0.770) levels in breast milk. Also, no significant association was found between DHA levels and saturated (*r* = 0.088, *p* = 0.502), monounsaturated (*r* = -0.039, *p* = 0.770) or polyunsaturated fatty acid levels (*r* = -0.178, *p* = 0.893). Yet, a significant negative correlation was found between DHA levels and n - 6:n - 3 ratio in breast milk (*r* = -0.503, *p* = 0.000).

### Correlation between dietary intakes and DHA levels in breast milk

According to the FFQ (Table 3), more than half of the participants (~ 55%) noted fish consumption only rarely or never, but ~ 60% of the participants consumed eggs at least twice a week. Vegetable oils were consumed every day or almost every day by ~ 81% of the participants. Milk was the most frequently used foodstuff – consumed everyday by approximately half of the participants. A significant correlation was found between fish, butter and avocado consumption and DHA levels in breast milk. Spearman’s correlation coefficients were 0.318 (*p* = 0.013), -0.283 (*p* = 0.028) and 0.342 (*p* = 0.007), respectively.

**Table 3:** Intake frequency (%) of food products and association with DHA levels in breast milk (n = 67)^a^

Food	Never	Seldom	Once a week	Twice a week	More than twice a week but not everyday	Every day	Correlation with DHA levels in breast milk (*n* = 60)
Fish	17.9	37.3	31.3	6.0	6.0	1.5	*r* = 0.318 (*p* = 0.013)
Crustaceans	43.3	49.3	6.0	1.5	0.0	0.0	*r* = 0.044 (*p* = 0.7395)
Eggs	13.4	14.9	11.9	23.9	28.4	7.5	*r* = -0.069 (*p* = 0.602)
Poultry, turkey meat	14.9	22.4	22.4	22.4	17.9	0.0	*r* = 0.072 (*p* = 0.585)
Pork	23.9	10.4	23.9	19.4	20.9	1.5	*r* = 0.077 (*p* = 0.559)
Beef	23.9	35.8	26.9	10.4	3.0	0.0	*r* = -0.006 (*p* = 0.966)
Meat products (sausages, nuggets, etc.)	31.3	35.8	4.5	19.4	6.0	3.0	*r* = -0.055 (*p* = 0.676)
Milk	19.4	11.9	3.0	3.0	13.4	49.3	*r* = 0.067 (*p* = 0.609)
Fermented dairy products (yoghurt, kefir, etc.)	23.9	17.9	14.9	9.0	14.9	19.4	*r* = 0.003 (*p* = 0.983)
Curd	19.4	19.4	13.4	23.9	11.9	11.9	*r* = -0.001 (*p* = 0.994)
Cheese	16.4	7.5	9.0	11.9	34.3	20.9	*r* = -0.103 (*p* = 0.432)
Sour cream	19.4	6.0	9.0	14.9	26.9	23.9	*r* = -0.236 (*p* = 0.070)
Cream	22.4	50.7	17.9	4.5	1.5	3.0	*r* = 0.006 (*p* = 0.965)
Milk desserts	26.9	31.3	10.4	22.4	6.0	3.0	*r* = -0.121 (*p* = 0.355)
Butter	17.9	9.0	4.5	13.4	23.9	31.3	*r* = -0.283 (p = 0.028)
Margarine	85.1	9.0	1.5	0.0	3.0	1.5	*r* = 0.031 (*p* = 0.815)
Fat spreads	97.0	1.5	0.0	0.0	0.0	1.5	*r* = 0.110 (*p* = 0.405)
Vegetable oils	1.5	3.0	4.5	10.4	46.3	34.3	*r* = -0.040 (*p* = 0.764)
Avocado	16.4	37.3	26.9	6.0	7.5	6.0	*r* = 0.342 (p = 0.007)
Nuts	9.0	31.3	19.4	9.0	17.9	13.4	r = 0.039 (*p* = 0.769)
Seeds	13.4	38.8	13.4	4.5	16.4	13.4	*r* = 0.178 (*p* = 0.173)

^a^FFQ covering the dietary habits of the last month prior to breast milk collection

The Kruskal-Wallis test with pairwise comparisons revealed that only mothers with fish intake at least twice a week had a significantly higher DHA levels in breast milk than non-fish eaters (*p* = 0.021) (Table 4).

**Table 4:** Association between fish intake and DHA levels in breast milk (*n* = 60)

Fish intake among participants	non/seldom (*n* = 34)	once a week (*n* = 18)	≥ 2 a week (*n* = 8)
Average DHA level in breast milk, %	0.32 ± 0.15^a^	0.34 ± 0.12^ab^	0.55 ± 0.24^b^

^a,b^– values with different letter is significantly different (*p* = 0.021)

According to the food diaries, the most frequently consumed fish species among the participants were salmon and herring, both reported by six and five women, respectively (Table 5). Two participants consumed two different fish species in one day and one participant consumed fish and caviar in one day.

**Table 5:** Marine food sources reported by participants in the three-day food diary (*n* = 60)

DHA source	Frequency, n
Salmon	6
Herring	5
Tuna	4
Cod	3
Brook trout	3
Trout	2
Carp	1
Sardines	1
Haddock	1
Grouper	1
Pike	1
White fish	1
Fish sticks (contains minced pollock)	3
Crustaceans	3
Caviar	1

The average ± SEM daily DHA intake according to the food diaries was 136 ± 26, 137 ± 33 and 178 ± 49 mg for the third, second and last day prior to sampling day, respectively and significantly correlated with DHA levels in the breast milk. Spearman’s correlation coefficients were 0.476, 0.550 and 0.540 for the third, second and last day prior to sampling day, respectively (*p* = 0.000 for all three days) (Table 6) and there was no statistically significant difference for DHA intake among the days (*p* = 0.643).

**Table 6:** Daily DHA intake among participants and association with DHA levels in breast milk (*n* = 60)

Dietary DHA intake	Third day prior to sampling	Second day prior to sampling	Last day prior to sampling
Average ± SEM^a^, mg	136 ± 26	137 ± 33	178 ± 49
Min, mg	0.00	0.00	0.00
Max, mg	1082	1439	2046
Correlation with DHA level in breast milk	*r* = 0.476 (*p* = 0.000)	*r* = 0.550 (*p* = 0.000)	*r* = 0.540 (*p* = 0.000)

^a^standard error of the mean

Only ten participants noted the use of marine oils – fish oil (*n* = 7), krill oil (*n* = 2) or algae oil (*n* = 1) in their food diaries. From all participants, two mothers combined fish and dietary supplement use on the same day, but 25 women did not report any intake of marine food or DHA supplements in the three-day food diary.

Linear regression was used to predict DHA levels in breast milk based on the FFQ records for fish intake. One of the DHA values (*n* = 60) was excluded from the linear regression as an outlier. It was established that fish intake significantly affects DHA level in breast milk, F(1,56) = 15.054, *p* = 0.000), and fish eating frequency accounted for 21.2% of the variability in breast milk DHA level. The regression equation was:

expected DHA level in breast milk = 0.245 + (0.070 x fish intake frequency).

## Discussion

Our obtained median DHA level in breast milk (0.30% of total fatty acids) corresponds to Jackson and Harris’ suggested target DHA value in breast milk, however, the optimal DHA level remains to be established [31]. An approximate DHA level of 0.30% in milk is achieved when a DHA intake of 200 mg is consumed – recommended value, rounded for ease of reference [31, 32]. Brenna and Lapillonne [32] have developed a regression analysis equation to calculate breast milk DHA level depending on dietary DHA intake using the data from Gibson et al. [33]:

breast milk DHA level (%, *w*/w) = 0.72 x maternal DHA intake (g per day) + 0.20.

Inserting our calculated average maternal DHA intakes into this equation, we obtain results, similar to the obtained median DHA level in breast milk from our analysis. Our acquired results from equation were – 0.30, 0.30 and 0.33% for the third, second and the last day prior to sampling day, respectively. Although daily DHA intake in our study was lower than recommended, the target DHA value in breast milk was reached.

Theoretical daily intake of DHA for infants referring to our obtained median DHA level would be ~ 98 mg, assuming that the standard diurnal breast milk intake is 798 g with an average fat content of 4.1% [34]. This is nearly (~ 98%) the recommended daily DHA intake for infants – 100 mg [9].

Our obtained median DHA level in breast milk was also similar to data obtained from Mediterranean countries like Spain (0.31–0.38%), Italy (0.35%), France (0.32%) and Nordic countries – Denmark (0.35%) and Iceland (0.30%) [19].

Our results for LA level in mature breast milk (11.50%) was slightly lower than the value of 12.67%, obtained combining data from The Netherlands, Caribbean Region, Jerusalem, Tanzania and Pakistan [35]. Also, our obtained median ALA level (1.00%) was noticeably higher than calculated in a study done by Nishimura et al. (0.09%) [21].

The level of ARA (0.30%) in our study was lower than observed by a meta-analysis of global data (0.47%) [19].

Our obtained ratio for n - 6:n - 3 fatty acids in breast milk (7:1) was twice as low as found for mature breast milk composition among Brazilian women (14:1) [22]. Nishimura et al. and Silva et al. suggest that a diet with low fish but high vegetable oil (particularly, soybean oil) consumption facilitates a higher n - 6:n - 3 ratio (above 10:1) [21, 22]. However, we were not able to demonstrate a significant correlation between vegetable oil or fish consumption frequency and the n - 6:n - 3 ratio in breast milk. Although there is no specified values for the n - 6:n - 3 ratio in the diet, the recommended proportion has been proposed as 4:1 [4, 36]. A very high n - 6:n - 3 ratio (~ 15:1) promotes the development of diseases, like cardiovascular disease, cancer (colorectal, breast), etc. [36].

There is evidence that the DHA level in breast milk has a positive correlation with intelligence quotient, whereas it has been shown that LA level has a negative correlation, indicating the importance of the fatty acid profile during the breastfeeding period [5, 37]. Our obtained ratio for DHA:LA (0.03:1) was similar to the mean ratio obtained analysing study data from 42 countries [38]. A higher DHA:LA ratio (~ 0.04:1–0.07:1) is linked to higher cognitive test score results [38], however, only one third of the participants from our study (*n* = 18) had a DHA:LA ratio of at least 0.04:1.

We found a significant correlation between fish consumption and DHA levels in breast milk which is consistent with results obtained from other studies [5, 24, 26]. However, some studies have failed to find this association [39]. It might depend on fish species most commonly consumed in a specific region or fish breeding (wild or farmed) conditions. For example, absolute DHA intake when consuming 100 g of farmed salmon is higher compared to eating the same quantity of wild salmon (0.88% versus 0.31%) [40]. This is linked to the higher fat content of farmed salmon – 12.3% versus 2.07% of fat [40]. In our study, the most commonly consumed fish species were salmon and herring that contain a high amount of DHA. Atlantic salmon (farmed) contains 1.104 g 100 g^- 1^ of DHA, whereas Atlantic herring (no information about breeding) contains 0.862 g 100 g^- 1^ of DHA [30]. Despite a lower fat content, tuna and pollock also can contribute to consumption of considerable amounts of DHA [40] – 0.196 mg 100 g^- 1^ and 0.350 mg 100 g^- 1^, respectively [30]. Quinn and Kuawa [17] concluded that an additional portion of fish consumed per week led to a 0.014% increase of DHA in breast milk, which is twice as high as we calculated. However, it should be noted that other foodstuff, like eggs and meat, also are substantial DHA sources [5, 32, 41].

We also observed that DHA levels in breast milk correlated with avocado and butter consumption. Avocados contain high amounts of monounsaturated fats (~ 9.80 g 100 g^- 1^), especially – oleic acid (9.07 g 100 g^- 1^) [30]. Butter mainly consists of saturated fat (57.05 g 100 g^- 1^), with the dominating fatty acid – palmitic acid (24.52 g 100 g^- 1^) [30]. Both fatty acids were the predominant fatty acids in breast milk samples in our study, however, no significant correlation between avocado and oleic acid levels or butter and palmitic acid levels in breast milk was found. Also, observed correlations were independent from fish intake frequency.

The average maternal daily DHA intake was calculated as 136 ± 26, 137 ± 33 and 178 ± 49 (SEM) mg for the third, second and last day prior to sampling day, which is lower than the recommended daily intake for DHA during lactation (~ 200 mg per day) [3, 4].

The relative proportion of DHA in breast milk in our study did not correlate with any of the maternal, or infants’ characteristics. Although, Quinn and Kuzawa have demonstrated that parity is a significant positive predictor for DHA levels in breast milk [17].

## Conclusions

Our obtained median DHA breast milk level (0.30 ± 0.18% of total fatty acids) corresponded to the target DHA value in breast milk, however the variance among participants was large (0.10 to 0.90%) and the total average daily DHA intake among participants was below the recommended daily intake (200 mg). DHA levels in breast milk were not affected by any of the evaluated maternal or infants’ characteristics, breastfeeding manner or milk expression method but in our study we established a positive association between fish consumption and breast milk DHA levels. However, fish consumption among breastfeeding women was low – more than half of participants were non-fish eaters or consumed them rarely.
